# Long-term studies of hantavirus reservoir populations in the southwestern United States: rationale, potential, and methods.

**DOI:** 10.3201/eid0501.990111

**Published:** 1999

**Authors:** J N Mills, T L Yates, T G Ksiazek, C J Peters, J E Childs

**Affiliations:** Centers for Disease Control and Prevention, Atlanta, Georgia 30333, USA. jum0@cdc.gov

## Abstract

Hantaviruses are rodent-borne zoonotic agents that cause hemorrhagic fever with renal syndrome in Asia and Europe and hantavirus pulmonary syndrome (HPS) in North and South America. The epidemiology of human diseases caused by these viruses is tied to the ecology of the rodent hosts, and effective control and prevention relies on a through understanding of host ecology. After the 1993 HPS outbreak in the southwestern United States, the Centers for Disease Control and Prevention initiated long-term studies of the temporal dynamics of hantavirus infection in host populations. These studies, which used mark-recapture techniques on 24 trapping webs at nine sites in the southwestern United States, were designed to monitor changes in reservoir population densities and in the prevalence and incidence of infection; quantify environmental factors associated with these changes; and when linked to surveillance databases for HPS, lead to predictive models of human risk to be used in the design and implementation of control and prevention measures for human hantavirus disease.


The following articles on hantavirus contain preliminary results of 3 years of longitudinal mark-release-recapture studies in the southwestern United States, provide an analysis of the sensitivity of the field techniques and statistical analyses for detecting changes in rodent population densities, and discuss the role of longitudinal studies in understanding reservoir host ecology as it relates to human disease. Although the data presented in the articles are overall standardized, methods used to extract information vary by research institution.

### Long-Term Studies of Hantavirus Reservoir Populations in the Southwestern United States: Rationale, Potential, and Methods

**James N. Mills, Terry L. Yates, Thomas G. Ksiazek, C.J. Peters, and James E. Childs**
Centers for Disease Control and Prevention, Atlanta, Georgia, USA

Hantaviruses are rodent-borne zoonotic agents that cause hemorrhagic fever with renal syndrome in Asia and Europe and hantavirus pulmonary syndrome (HPS) in North and South America. The epidemiology of human diseases caused by these viruses is tied to the ecology of the rodent hosts, and effective control and prevention relies on a thorough understanding of host ecology. After the 1993 HPS outbreak in the southwestern United States, the Centers for Disease Control and Prevention initiated long-term studies of the temporal dynamics of hantavirus infection in host populations. These studies, which used mark-recapture techniques on 24 trapping webs at nine sites in the southwestern United States, were designed to monitor changes in reservoir population densities and in the prevalence and incidence of infection; quantify environmental factors associated with these changes; and when linked to surveillance databases for HPS, lead to predictive models of human risk to be used in the design and implementation of control and prevention measures for human hantavirus disease.

Hantaviruses (genus *Hantavirus,* family Bunyaviridae) are rodent-borne zoonotic agents that cause mild to severe hemorrhagic fevers throughout most of Europe, Asia, and the Americas. The epidemiology of these hemorrhagic fevers is largely defined by the distribution and ecology of the rodent hosts of the viruses. Hantaviruses have been identified at a dramatically increased rate in recent years; some 30 hantaviruses are now recognized throughout the world (1,2). With very few exceptions, each virus is associated with a single primary rodent host of the family Muridae. The rodent, in which the virus establishes a chronic infection, sheds infectious virus into the environment in urine, feces, and saliva (3,4); these characteristics are key to the transmission of the virus, both to humans (most frequently by inhalation of infectious aerosols [5]) and among rodents (frequently by aggressive encounters and biting [6,7]).

Human diseases due to hantaviruses, which have been recognized at least since World War I and probably occurred much earlier (8), were unknown in the Americas until recently. Although *Rattus norvegicus* infected with Seoul virus is common in many cities throughout the Americas (9), human disease associated with a

Address for correspondence: James N. Mills, Centers for Disease Control and Prevention, 1600 Clifton Road, Mail Stop G14, Atlanta, GA 30333, USA; fax: 404-639-1118; e-mail: jum0@cdc.gov.

rat-borne hantavirus was not documented in a U.S. city until 1994 (10). Prospect Hill virus, an indigenous North American hantavirus, was isolated from the meadow vole (*Microtus pennsylvanicus*) as early as 1982 (11) but has never been associated with human disease.

Since 1993, when hantavirus pulmonary syndrome (HPS) was recognized and its etiologic agent, Sin Nombre virus (SNV), was isolated and associated with the deer mouse (*Peromyscus maniculatus*) (12,13), at least 20 New-World hantaviruses, all associated with the same group of indigenous American rodents (family Muridae, subfamily Sigmodontinae) have been described, and HPS has been diagnosed from Canada to Patagonia. The severity (approximately 50% death rate) and wide geographic distribution of this rodent-borne zoonotic disease has prompted intensive collaboration between public health investigators and ecologists to elucidate the ecologic and epizootiologic features of infection in host populations and the factors that lead to human infection.

Because no specific treatment is yet available, prevention measures are essential in decreasing HPS-related illness and death. Developing effective preventive measures requires a detailed knowledge of the ecology and epizootiology of hantavirus infection in reservoir populations and the specific situations and mechanisms that result in the transfer of hantaviruses from hosts to humans.

### Reservoir Studies

Reservoir studies, whose role in understanding, controlling, and preventing human disease has been reviewed, have resulted in a series of research goals that may facilitate the collection of data concerning reservoir ecology, a subject pertinent to human health (14). The first goal is to identify the reservoir host; others are a) to determine the area in which the disease may be endemic by identifying the geographic range of the host and the range of infection by the pathogen within the host range; b) to more precisely define relative risk to humans by determining the distribution of the host and pathogen among distinct habitats regionally; c) to investigate potential mechanisms of pathogen transmission within host populations by conducting cross-sectional surveys to define the prevalence of infection among various subpopulations of the host (e.g., male versus female, juvenile versus adult); d) to conduct long-term prospective studies to explain the temporal patterns of infection in host populations; and e) to integrate the data from these studies in a predictive model that will allow early identification of specific times and places where conditions can increase rodent populations or infection in rodent populations and elevate the risk for human disease. This model could be used to minimize the incidence of human disease through public education, habitat modification, or reservoir control.

Investigations of HPS cases in the United States have resulted not only in studies of the deer mouse and SNV but also in the identification of three additional host-virus relationships that maintain hantaviruses responsible for human disease (New York virus carried by the white-footed mouse, *Peromyscus leucopus* [Figure 1] [15]; Black Creek Canal virus carried by the cotton rat, *Sigmodon hispidus* [16]; and Bayou virus carried by the rice rat, *Oryzomys palustris* [17]). Most HPS cases in the United States have been caused by SNV.

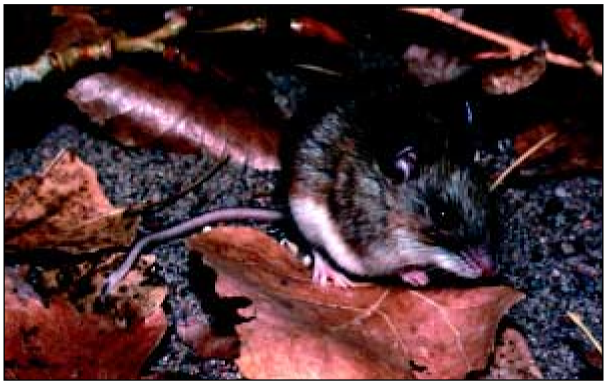

Figure 1. White-footed mouse (*Peromyscus leucopus*). Photo by R.B. Forbes, Mammal Image Library of the American Society of Mammalogists.

Intensive studies of deer mouse populations have addressed most of the proposed goals. One of the most common and most extensively studied small mammals in North America, the deer mouse has a well-known geographic distribution (18). Antibody screening of deer mouse populations throughout North America has provided evidence of SNV infection throughout most of the species' range (Ksiazek et al., unpub. data). Regional studies have shown differences in the prevalence of hantavirus infection among deer mouse populations in different habitats and helped define the varying disease risk to humans in these habitats (7). Finally, studies of the age- or size-specific prevalence of hantavirus infection among reservoir populations have shown that SNV, and other hantaviruses, are transmitted horizontally within reservoir populations, and that one important specific mechanism of transfer may be aggressive encounters and bites, most frequently among male animals (6,7,19). Although these cross-sectional studies have increased our understanding of host-virus ecology as it relates to human disease, they have not explained the temporal dynamics of host-virus ecology nor have they identified the environmental factors associated with these dynamics; only long-term prospective studies can provide this additional information.

### Long-Term Studies

Long-term studies, widely regarded by ecologists as indispensable for understanding the temporal dynamics of vertebrate communities (20), are especially useful for assessing the effects of rare events (e.g., El Niño southern oscillation) and for detecting and observing processes that unfold slowly in communities or populations (e.g., establishment or disappearance of a reservoir species from part of its range; changes in reservoir population density; changes in community composition; introduction or extinction of a pathogen in a specific host population; and changes in the incidence or prevalence of infection within the host population).

Long-term studies of reservoir populations have helped elucidate the temporal dynamics of hantavirus infection in host populations for Seoul and Prospect Hill viruses (21) and identify characteristics of reservoir ecology associated with outbreaks of human disease. The numbers of cases of hemorrhagic fever with renal syndrome due to Puumala virus in Scandinavia and Argentine hemorrhagic fever due to Junín virus (an arenavirus with many epidemiologic similarities to hantaviruses) were correlated with cyclic changes in the density of reservoir host populations (22,23). Increases in population density are associated with improved reproductive success and survivorship that may be due to improved habitat. Changes in the environment may be associated with favorable weather patterns, accelerated vegetation growth, and availability of plant and small-animal foods (14,23). The 1993 HPS outbreak in the southwestern United States may have resulted from improvements in the quality of deer mouse habitat caused by the 1991-92 El Niño southern oscillation (24). When the environmental variables associated with increasing reservoir population densities are identified and quantified, a key component of a predictive model of human risk will be in place.

Despite their importance and utility, long-term studies of reservoir populations associated with zoonotic agents are rare. By definition, they require stable funding for many years, they are labor intensive, expensive, and may not produce significant results in the short term. The periodic shifts in environmental conditions that change host populations and increase risk for human disease may take many years.

The most common method for conducting long-term studies of small-mammal populations is the mark-release-recapture (MRR) technique. Animals trapped live on permanent trapping plots are measured, sampled (blood or oral swab), identified with a permanent mark or number, and released at the exact site of capture. The trapping plots are operated at predetermined intervals for several days. Animals recaptured in subsequent trappings are measured and sampled again so that changes in numbers of animals, body growth rates, movement, reproductive condition, and infection status can be monitored. Environmental variables such as weather conditions and vegetative cover also may be monitored on the trapping plots. Control plots, where invasive procedures are minimized, may be necessary for determining or correcting for the influence of sampling methods on animal survival or population size.

After the 1993 HPS outbreak, the Centers for Disease Control and Prevention (CDC) established a network of hantavirus and rodent monitoring sites in the southwestern United States to 1) monitor and quantify the seasonal and year-to-year changes in host population density and the prevalence and incidence of hantavirus infection, 2) identify and quantify the biotic and abiotic environmental factors associated with and likely influencing these dynamics, 3) identify mechanisms of virus transmission within reservoir populations, and 4) identify and measure any effects of infection on individuals and populations of the host.

The studies should lead to predictive models of human risk for hantavirus infection and should facilitate prevention and control of human hantavirus disease.

### Study Sites

Arizona, Colorado, and New Mexico were chosen as the general study area because of their high numbers of HPS cases at the time the study was being designed. In addition, four sigmodontine rodent species identified as hantavirus reservoirs inhabit at least parts of the three-state area (*P. maniculatus, P. boylii, Reithrodontomys megalotis,* and *S. hispidus*). Longitudinal MRR studies are being conducted on 24 trapping webs at nine sites in the three states (Figure 2): 10 webs at four sites in New Mexico, operated by the University of New Mexico; six webs at three sites in Colorado, operated by Colorado State University; four webs at one site in northern Arizona, operated by Yavapai College; and four webs at one site in southern Arizona, operated by the University of Arizona. Site selection criteria included presence of populations of *Peromyscus* spp. and evidence of infection by SNV or related viruses in these populations. The location of each trapping web was fixed precisely by global positioning system technology. A sketch of each trapping web site, including a description of the vegetation, was prepared.

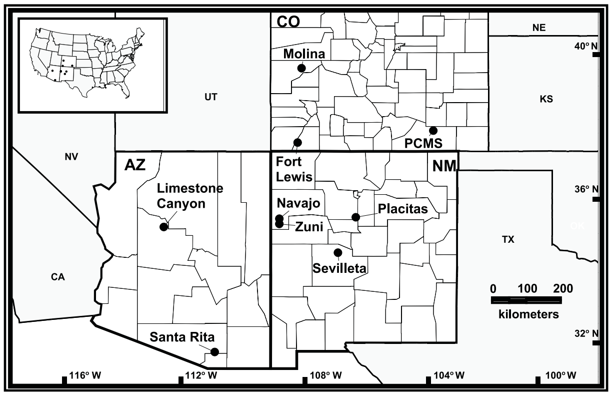

Figure 2. Geographic locations of nine sites where mark-release-recapture webs are being operated to study rodent reservoirs of hantaviruses in a three-state area of the southwestern United States. PCMS=Pinyon Canyon Maneuver Site (U.S. Army).

### Placing Permanent Trapping Webs

Small-mammal populations were monitored through the use of permanent trapping webs (25) (Figure 3A). Each web covered 3.14 ha and contained 12 100-m transects radiating from a central point and resembling the spokes of a wheel (Figure 3B). Each web contained 148 Sherman (8 x 9 x 23 cm; H.B. Sherman Trap Company, Tallahassee, FL) and 24 Tomahawk (14 x 14 x 40 cm; Tomahawk Live Trap Company, Tomahawk, WI) live-capture traps, at 12 trap stations along each radiating spoke. The first four trap stations were at 5-m intervals and the remaining eight at 10-m intervals. Four Sherman traps were placed around the central point. In addition to a Sherman trap, one Tomahawk trap was placed at trap stations 7 and 12 in each radiating arm (Figure 3B). Two to four webs were located at each sampling site. At least one web at each site was designated a control web. At this web, rodent populations were monitored but not sampled by blood and oral swab collection so that the effects of sampling on small-mammal survivorship could be assessed. At the remaining webs, virus activity in small-mammal populations was monitored through monthly blood and oral swab samples from captured animals. After the second year of the study, the purpose of the control webs was achieved, so sampling of captured small mammals from these webs was initiated (25,26).

### Trapping Schedules

All trapping web sites were visited monthly, except those in Colorado, which were visited every 6 weeks, as weather permitted. Webs were operated for 3 consecutive nights on each trapping occasion, generally coinciding with the new moon. Traps were set out in the evening of the first day and baited with peanut butter and rolled oats, cracked corn, or mixed grain. In cold weather, cotton or polyester fiberfill was placed in the traps to provide nesting material and reduce trap-associated deaths.

Captured rodents were collected, transported, and sampled according to standardized procedures (27,28). Briefly, traps were checked for captures early each morning. Investigators wearing rubber gloves collected the traps containing captured animals, labeled them with the web and trap station number, and placed them in double plastic bags for transport to a centralized outdoor processing station. Before

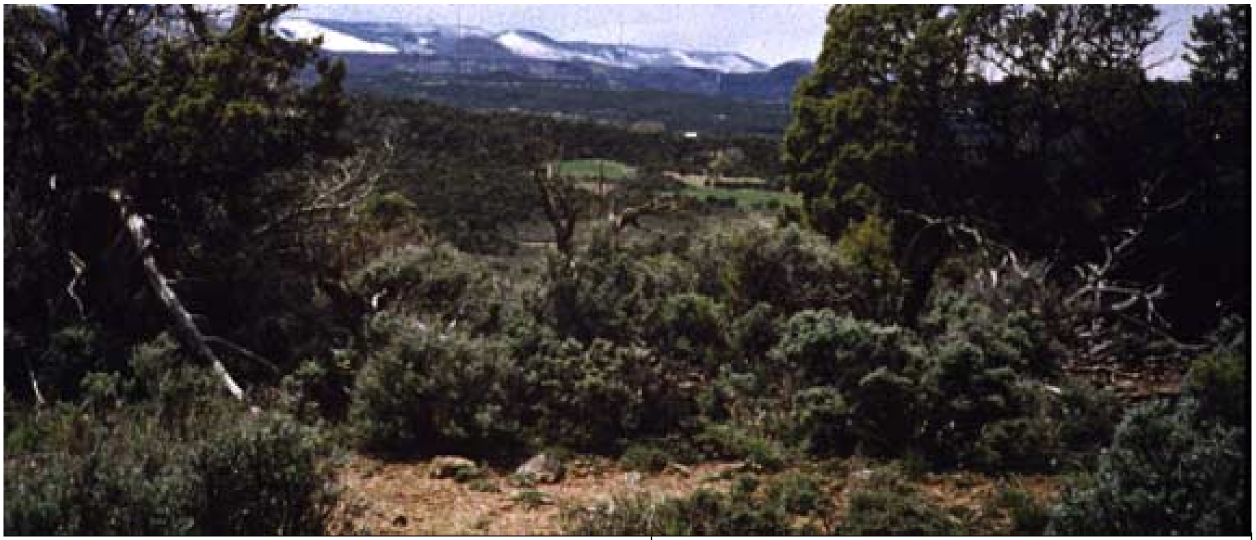

A

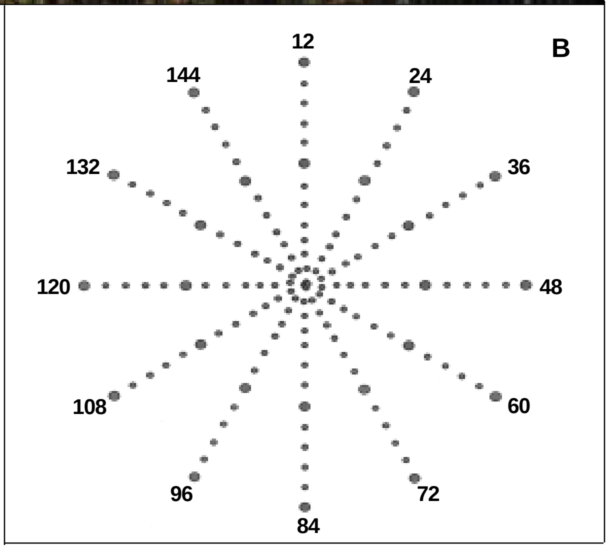

Figure 3. A. Characteristics of landscape and vegetation near Fort Lewis trapping web "A," southwestern Colorado.
Photo courtesy of C. Calisher.
B. Schematic representation of a trapping web showing the relative locations of the 148 trap stations. Small circles indicate the location of one Sherman trap, larger circles, one Sherman plus one Tomahawk trap. Diameter of the web was 200 m. After Parmenter et al. (25).

opening the bags containing captured small mammals, investigators donned protective clothing, including latex gloves, disposable surgeon's gowns, and respirators fitted with HEPA filters. Each captured animal was processed individually. The animal was first shaken from the trap into a plastic bag containing cotton or gauze soaked with inhalant anesthesia (methoxyflurane, Pitman-Moore, Mundelein, IL; or isoflurane, Abbott Laboratories, North Chicago, IL). To prevent potential cross-infection between animals, each was anesthetized in a clean plastic bag, and the anesthesia-soaked cotton was contained in a tea strainer that allows diffusion of the anesthesia, yet between animals can be wiped with a disinfectant. In one case, investigators used a specially adapted "nose cone" for anesthesia (Abbott et al., this issue, pp. 102-112). After being anesthetized, the animal was removed from the bag and placed on a clean surface. A standardized form was used at all trapping sites to collect the following data (28): unique capture number; date of capture; exact location of capture on the trapping web (trap station number); ear tag number; fate (first capture, recapture [different trapping session], or repeater [within same 3-day trapping session]); species; age (juvenile, subadult, or adult); mass; lengths of tail plus body, tail only, ear, and right hind foot; reproductive status including position of the testes (scrotal or abdominal) for males and condition of the vagina (closed or perforate) and description of the nipples (enlarged or small, lactating or not) for females; and the presence or absence of scars or wounds. For animals from the sampling webs, oral swabs were taken with Dacron-tipped applicators cut with scissors at the level of the Dacron and inserted into 0.5 ml of virus medium (phosphate-buffered saline containing 20% fetal bovine serum, 2% penicillin and streptomycin, and 0.1% Fungizone) in a 2-ml cryovial. Approximately five drops of whole blood were collected into a second cryovial by a capillary tube inserted into the retro-orbital capillary plexus. Whole blood and oral swab samples were immediately placed in liquid nitrogen or on dry ice until transferred to -70°C freezers for storage. Animals recaptured on days 2 or 3 of the trapping session were not bled a second time to avoid trauma. Animals newly captured were marked with a uniquely numbered ear tag (some smaller animals were marked by toe clipping). The animal was then replaced in the original trap or in a clean, ventilated one-quart, screw-capped jar, and was allowed to recover fully from the effects of anesthesia and was released at the exact site of capture. A clean, baited trap was replaced at the site, and the original trap was returned to the processing site to be decontaminated before reuse. Animals from the control webs were treated similarly, except that blood and oral swab samples were not taken.

Investigators recorded environmental data, including a general description of the vegetation and depending on resources available, more detailed descriptions of vegetation at individual webs and weather conditions during the trapping session (detailed rainfall and temperature data were available from meteorologic stations near each trapping site).

### Laboratory Analysis

Serologic testing was conducted at CDC, Atlanta, or at the Arthropod-Borne and Infectious Diseases Laboratory, Colorado State University, Fort Collins, CO, USA. Samples of whole blood were tested for antibody reactive with SNV recombinant nucleocapsid protein antigen by enzyme-linked immunosorbent assay according to a standardized protocol (29). Briefly, blood specimens were initially diluted 1:25 in 5% skim milk in 0.01 M phosphate-buffered saline with 0.5% Tween-20 and subsequently diluted to 1:100 through 1:6,400 in fourfold dilutions in microtiter plates. Samples were tested against the recombinant nucleocapsid antigen and a recombinant control antigen (29). A conjugate mix of anti-*Rattus norvegicus* and anti-*Peromyscus leucopus* (heavy and light chains) immunoglobulin G (IgG) (Kirkegaard and Perry, Gaithersburg, MD) was used to detect bound immunoglobulin. Adjusted optical densities (OD) for each dilution were calculated by subtracting the $OD_{410}$ of the control antigen from the $OD_{410}$ of the SNV antigen. Titers were assigned on the basis of an adjusted OD value exceeding 0.20 for each dilution. A second measure consisting of the sum of the adjusted OD values for all four dilutions was also calculated. Serum specimens were considered SNV-positive if their titer was 1:400 or their sum-adjusted OD was 0.95. The cut-off values were determined by assessment of rodents found to be SNV-positive by several serologic tests during the initial investigation of the 1993 outbreak (13) and have been reassessed periodically among large populations of rodents collected in North and South America. Antibodies to other North American hantaviruses are cross-reactive with SNV antigen. This assay would detect (but not distinguish among) infections by New York virus (from the white-footed mouse [15]), Prospect Hill-like viruses (from arvicoline rodents [30]), El Moro Canyon virus (from the Western harvest mouse [31]), Black Creek Canal virus (from the cotton rat [16]), and Bayou virus (from the rice rat [17]).

At this writing, analyses on oral swab specimens (antigen or antibody detection, polymerase chain reaction) have not been conducted. Blood and oral swab samples are archived in -70°C freezers at CDC, Atlanta, and at the Museum of Southwestern Biology, Albuquerque, New Mexico.

### Acknowledgments

We are indebted to many investigators at CDC for laboratory analyses and database management. Special thanks to R. Meyer, M.L. Martin, V. Semenova, G. Gallucci, M. Curtis, A.J. Williams, and P. Stockton in the laboratory; B. Farrar in the stock rooms; and L. Morgan and K. Schmidt at the computer. K. Wagoner, K. Colbert, and B. Ellis prepared the figures. K. Abbott, C. Calisher, B. Ellis, and M. Morrison provided helpful comments on an earlier version of the manuscript.

Dr. Mills is chief of the Medical Ecology Unit, Special Pathogens Branch, Division of Viral and Rickettsial Diseases, CDC. His research interests include zoonotic diseases, specifically host-pathogen evolution and interactions.

### References

1. Schmaljohn CS, Hjelle B. Hantaviruses: a global disease problem. Emerg Infect Dis 1997;3:95-104.

2. Peters CJ, Mills JN, Spiropoulou C, Zaki SR, Rollin PE. Hantaviruses. In: Guerrant RL, Walker DH, Weller PF, editors. Tropical infectious diseases: principles, pathogens, and practice. New York: W.B. Saunders. In press 1999.
3. Lee HW, French GR, Lee PW, Baek LJ, Tsuchiya K, Foulke RS. Observations on natural and laboratory infection of rodents with the etiologic agent of Korean hemorrhagic fever. Am J Trop Med Hyg 1981;30:477-82.
4. Hutchinson KL, Rollin PE, Peters CJ. Pathogenesis of a North American hantavirus, Black Creek Canal virus, in experimentally infected *Sigmodon hispidus*. Am J Trop Med Hyg 1998;59:58-65.
5. Tsai TF. Hemorrhagic fever with renal syndrome: mode of transmission to humans. Lab Anim Sci 1987;37:428-30.
6. Glass GE, Childs JE, Korch GW, LeDuc JW. Association of intraspecific wounding with hantaviral infection in wild rats (*Rattus norvegicus*). Epidemiol Infect 1988;101:459-72.
7. Mills JN, Ksiazek TG, Ellis BA, Rollin PE, Nichol ST, Yates TL, et al. Patterns of association with host and habitat: antibody reactive with Sin Nombre virus in small mammals in the major biotic communities of the southwestern United States. Am J Trop Med Hyg 1997;56:273-84.
8. McKee KT Jr, LeDuc JW, Peters CJ. Hantaviruses. In: Belshe RB, editor. Textbook of human virology. St Louis (MO): Mosby Year Book; 1991. p. 615-32.
9. LeDuc JW, Smith GA, Childs JE, Pinheiro FP, Maiztegui JI, Niklasson B, et al. Global survey of antibody to Hantaan-related viruses among peridomestic rodents. Bull World Health Organ 1986;64:139-44.
10. Glass GE, Watson AJ, LeDuc JW, Childs JE. Domestic cases of hemorrhagic fever with renal syndrome in the United States. Nephron 1994;68:48-51.
11. Lee P-W, Amyx HL, Gajdusek DC, Yanagihara RT, Goldgaber D, Gibbs CJ. New haemorrhagic fever with renal syndrome-related virus in indigenous wild rodents in United States. Lancet 1982;2:1405.
12. Nichol ST, Spiropoulou CF, Morzunov S, Rollin PE, Ksiazek TG, Feldmann H, et al. Genetic identification of a hantavirus associated with an outbreak of acute respiratory illness. Science 1993;262:914-7.
13. Childs JE, Ksiazek TG, Spiropoulou CF, Krebs JW, Morzunov S, Maupin GO, et al. Serologic and genetic identification of *Peromyscus maniculatus* as the primary rodent reservoir for a new hantavirus in the southwestern United States. J Infect Dis 1994;169:1271-80.
14. Mills JN, Childs JE. Ecological studies of rodent reservoirs: their relevance for human health. Emerg Infect Dis 1998;4:529-37.
15. Song JW, Baek LJ, Gajdusek DC, Yanagihara R, Gavrilovskaya I, Luft BJ, et al. Isolation of pathogenic hantavirus from white-footed mouse (*Peromyscus leucopus*) [letter]. Lancet 1994;344:1637.
16. Rollin PE, Ksiazek TG, Elliott LH, Ravkov EV, Martin ML, Morzunov S, et al. Isolation of Black Creek Canal virus, a new hantavirus from *Sigmodon hispidus* in Florida. J Med Virol 1995;46:35-9.
17. Ksiazek TG, Nichol ST, Mills JN, Groves MG, Wozniak A, McAdams S, et al. Isolation, genetic diversity and geographic distribution of Bayou virus (Bunyaviridae: *Hantavirus*). Am J Trop Med Hyg 1997;57:445-8.
18. Carleton MD. Systematics and evolution. In: Kirkland GL, Layne JN, editors. Advances in the study of *Peromyscus* (Rodentia). Lubbock (TX): Texas Tech University Press; 1989. p. 7-141.
19. Glass GE, Livingstone W, Mills JN, Hlady WJ, Fine JB, Rollin PE, et al. Black Creek Canal virus infection in *Sigmodon hispidus* in southern Florida. Am J Trop Med Hyg 1998;59:699-703.
20. Cody ML. Introduction to long-term community ecological studies. In: Cody ML, Smallwood JA, editors. Long-term studies of vertebrate communities. San Diego: Academic Press; 1996. p. 1-15.
21. Childs JE, Glass GE, Korch GW, LeDuc JW. Prospective seroepidemiology of hantaviruses and population dynamics of small mammal communities of Baltimore, Maryland. Am J Trop Med Hyg 1987;37:648-62.
22. Niklasson B, Hornfeldt B, Lundkvist A, Bjorsten S, LeDuc J. Temporal dynamics of Puumala virus antibody prevalence in voles and of nephropathia epidemica incidence in humans. Am J Trop Med Hyg 1995;53:134-40.
23. Mills JN, Ellis BA, McKee KT, Calderón GE, Maiztegui JI, Nelson GO, et al. A longitudinal study of Junín virus activity in the rodent reservoir of Argentine hemorrhagic fever. Am J Trop Med Hyg 1992;47:749-63.
24. Parmenter RR, Brunt JW, Moore DI, Ernest S. The hantavirus epidemic in the Southwest: rodent population dynamics and the implications for transmission of hantavirus-associated adult respiratory distress syndrome (HARDS) in the Four Corners region. Publication No.: 41. Albuquerque (NM): University of New Mexico; 1993. Sevilleta Long-Term Ecological Research Site.
25. Parmenter CA, Yates TL, Parmenter RR, Mills JN, Childs JE, Campbell ML, et al. Small mammal survival and trapability in mark-recapture monitoring programs for hantavirus. J Wildl Dis 1998;34:1-12.
26. Swann DE, Kuenzi AJ, Morrison ML, DeStefano S. Effects of sampling blood on survival of small mammals. Journal of Mammalogy 1997;78:908-13.
27. Mills JN, Yates TL, Childs JE, Parmenter RR, Ksiazek TG, Rollin PE, et al. Guidelines for working with rodents potentially infected with hantavirus. Journal of Mammalogy 1995;76:716-22.
28. Mills JN, Childs JE, Ksiazek TG, Peters CJ, Velleca WM. Methods for trapping and sampling small mammals for virologic testing. Atlanta (GA): U.S. Department of Health and Human Services; 1995.
29. Feldmann H, Sanchez A, Morzunov S, Spiropoulou CF, Rollin PE, Ksiazek TG, et al. Utilization of autopsy RNA for the synthesis of the nucleocapsid antigen of a newly recognized virus associated with hantavirus pulmonary syndrome. Virus Res 1993;30:351-67.
30. Yanagihara R, Daum CA, Lee PW, Baek LJ, Amyx HL, Gajdusek DC, et al. Serological survey of Prospect Hill virus infection in indigenous wild rodents in the USA. Trans R Soc Trop Med Hyg 1987;81:42-5.
31. Hjelle B, Chavez-Giles F, Torrez-Martinez N, Yates T, Sarisky J, Webb J, et al. Genetic identification of a novel hantavirus of the harvest mouse *Reithrodontomys megalotis*. J Virol 1994;68:6751-4.
